# Endovascular Management of Superior Mesenteric Artery (SMA) Aneurysm – Adequate Access is Essential for Success – Case Report

**DOI:** 10.12659/PJR.901935

**Published:** 2017-07-13

**Authors:** Lovro Tkalčić, Berislav Budiselić, Miljenko Kovačević, Siniša Knežević, Slavica Kovačić, Damir Miletić, Vjekoslav Tomulić, Dimitrij Kuhelj

**Affiliations:** 1 Department of Radiology, Clinical Hospital Centre Rijeka, Rijeka, Croatia; 2 Department of Vascular Surgery, Clinical Hospital Centre Rijeka, Rijeka, Croatia; 3 Department of Cardiology, Clinical Hospital Centre Rijeka, Rijeka, Croatia; 4 Clinical Radiology Institute, University Medical Centre Ljubljana, Ljubljana, Slovenia

**Keywords:** Aneurysm, Endovascular Procedures, Mesenteric Artery, Superior

## Abstract

**Background:**

An aneurysm of the superior mesenteric artery (SMA) with a diameter of 2.2 cm was found incidentally on an ultrasound (US) examination in a 26-year-old woman. The only known risk factor was an intracranial aneurysm that was found on her grandmother’s autopsy. Based on pregnancy planning and the current literature, endovascular management with a covered stent was proposed.

**Case Report:**

Self-expandable, covered stent (Bard, Fluency^®^) was implanted using a single transfemoral approach. A stiff guidewire and a large sheath distorted the anatomy, which resulted in an incomplete aneurysmal neck covering. In the absence of additional covered stents, the procedure was terminated. Two weeks later, computed tomographic angiography (CTA) confirmed persistent aneurysmal perfusion due to the incomplete neck coverage. A multidisciplinary board opted for a second endovascular attempt, this time with a longer covered stent via the transaxillary approach in order to reduce anatomical distortion. Balloon, expandable, cobalt-chrome covered stent (Jotec, E-ventus BX^®^) was implanted in the SMA, covering the aneurysmal neck and overlapping the previously implanted covered stent. Angiography confirmed a complete exclusion of the aneurysm.

A control US performed three weeks later confirmed a patent covered stent and complete aneurysmal exclusion. There was a mild median nerve damage periprocedurally that resolved in three months. The most recent US control examination, performed eleven months after the procedure, showed an excluded aneurysm and a patent covered stent. There were no clinical signs of bowel ischaemia during the follow-up period.

**Conclusions:**

Endovascular management of SMAA proved to be safe and efficient. The “access from above” is probably safer and should be considered in the majority of cases with acceptable sizes of access vessels. Mid-term results in our patient are good and life-long follow-up is planned to prevent late complications.

## Background

Visceral artery aneurysms (VAA) are relatively rare, accounting for 5–8% of all aneurysms, and have an annual incidence between 0.01–0.2% [1]. Superior mesenteric artery aneurysms (SMAAs) are the third most common true visceral aneurysms, often affecting the proximal 5 cm of the artery [2] and accounting for 14% of all VAAs [3]. Aneurysmal degeneration of the superior mesenteric artery occurs infrequently, but when it does, mesenteric ischaemia or rupture may result [4], which poses a potentially life-threatening condition [3]. In about 25% of cases, VAAs present as surgical emergencies [1], although the course of the disease is often asymptomatic. VAAs are an incidental finding in almost half of patients [5]. Around 22% of patients with VAAs are diagnosed after rupture. Variable clinical manifestations pose the risk of misdiagnosis and unwarranted treatment with high mortality – 8.5–25% [6,7].

The diagnosis of SMAA is sometimes based on an abdominal ultrasound examination, although a precise detection is often made by computed tomographic angiography (CTA) or magnetic resonance (MR). Calcified wall on x-rays might raise the suspicion of an aneurysm. Changes in the surrounding organs and tissues (signs of chronic pancreatitis etc.) might reveal the cause of the condition. Additional vascular changes might be discovered, and factors influencing management should be determined by evaluating clinical symptoms, laboratory findings and imaging (aneurysmal size, neck, access vessels etc.).

## Case Report

An abdominal US examination revealed an SMAA of unknown aetiology in an asymptomatic 26-year-old woman. Initial CTA confirmed the presence of an aneurysm with a diameter of 2.2 cm, originating from the proximal part of the superior mesenteric artery (SMA). Clinical workup included a detailed history, physical examination and blood analysis, including complete blood cell count, coagulation studies and biochemical analysis. There was no history of infectious or inflammatory diseases and no history of trauma; pancreatitis, alcohol abuse, liver disease and neoplasm were also excluded. The only known risk factor was a history of an intracranial aneurysm that found on her grandmother’s autopsy.

After a multidisciplinary consensus, taking into consideration patient’s age, pregnancy planning, aneurysmal size, location and literature data, endovascular management with a covered stent was proposed. Informed consent from the patient was obtained before the procedure.

**Figure 1:**
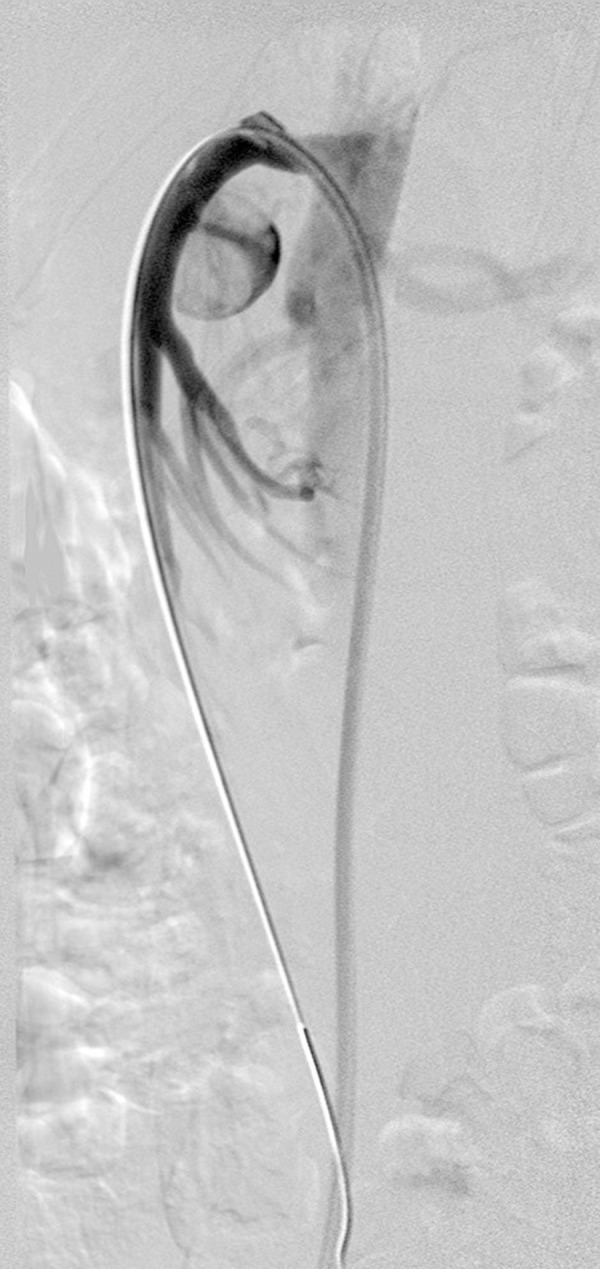
DSA confirmed aneurysm originating from the proximal part of SMA.

**Figure 2:**
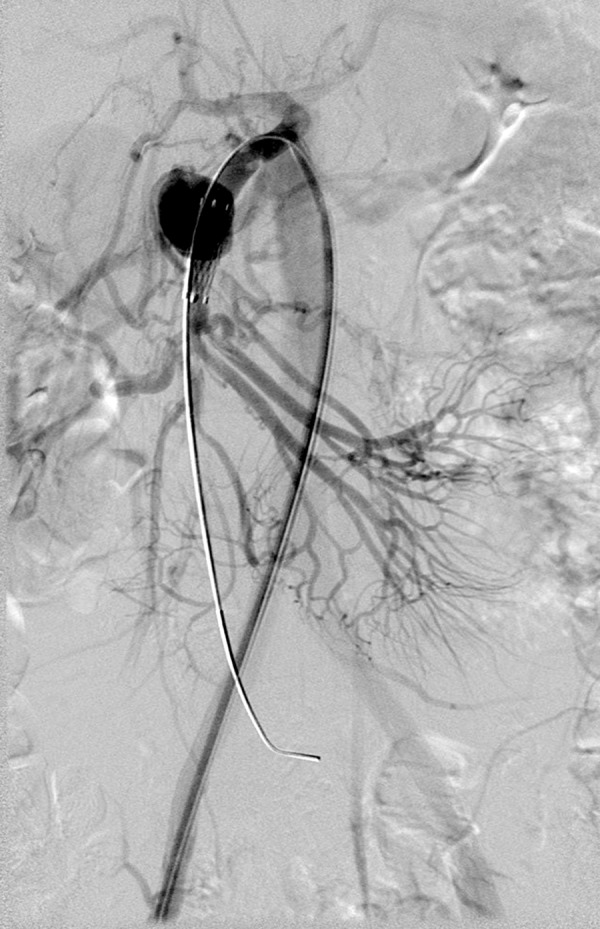
Control DSA showed persistent aneurysmal sack filling after implantation of self-expandable covered stent due to incomplete aneurysmal neck covering.

Due to a large size of the introducer sheath (9 Fr), required for the available covered stent implantation (Bard, Fluency^®^, 8×20 mm), the femoral approach was chosen. Digital subtraction angiography (DSA) confirmed previous findings (Figure 1). Selective SMA catheterisation was performed with the use of a curved guiding catheter and a stiff guidewire (Amplatz stiff^®^, Cook) that was placed distally in a branch of the SMA. Heparin (5000 IU) was administered into the SMA. An introducer sheath (9 Fr, 55 cm, Brite tip^®^, Cordis) was advanced into the SMA ostium and a self-expandable covered stent was implanted. A control DSA showed persistent aneurysmal sack filling (Figure 2). Single femoral approach, a stiff guidewire and a large sheath distorted the anatomy, resulting in an incomplete aneurysmal neck covering. Alternatively, only a rigid, stainless steel covered stent was available and therefore the procedure was terminated. The patient was discharged from the hospital on the following day without clinical signs of complications.

Two weeks later, CTA confirmed persistent aneurysmal perfusion due to incomplete neck coverage; the patient was still asymptomatic.

A multidisciplinary board consisting of a vascular a surgeon, cardiologist and interventional radiologists decided to opt for a second endovascular intervention. A longer covered stent with lower crossing profile was selected; we decided to preserve the stump of the SMA for a potential conversion to open surgery.

**Figure 3:**
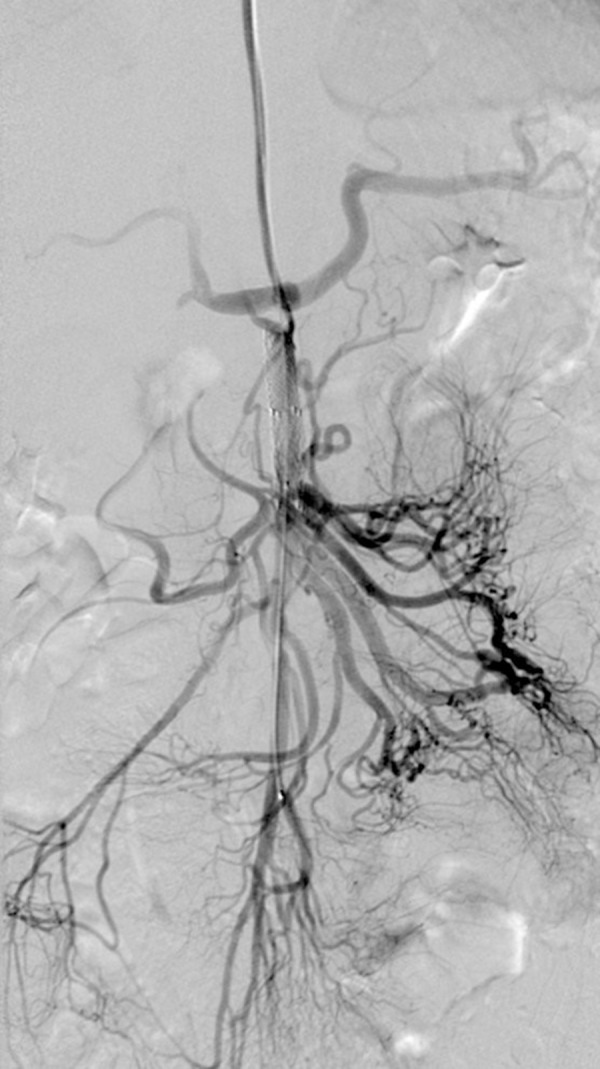
On second attempt, balloon, expandable covered stent was placed in the SMA covering the aneurysmal neck – DSA confirmed complete exclusion of the aneurysm.

A left transaxillary approach was used for anatomy preservation and an 8-Fr straight guiding catheter (55 cm, Brite tip^®^, Cordis) was introduced into the SMA. Balloon, expandable cobalt-chromium covered stent (E-ventus BX^®^, Jotec, 8×50 mm) was placed in the SMA, covering the aneurysmal neck (Figure 3). A control DSA confirmed a complete exclusion of the aneurysm.

Complete aneurysmal exclusion and a patent covered stent were confirmed on control US three weeks later, and the patient was free of any signs of bowel ischaemia. Due to the transaxillary approach and 8-Fr sheath used, there was a mild median nerve damage periprocedurally that resolved in the following three months. No additional clinical signs of complications were noted during the follow-up period and an control US examination, performed 11 months later, showed a completely excluded aneurysm and a patent covered stent.

## Discussion

We report a case of challenging endovascular treatment of a rare SMA aneurysm in a young patient. As seen in our case, SMAAs are often asymptomatic and incidental.

Complications of endovascular treatment are not uncommon, since 25% of patients (9 patients) in a patient series [5] had persistent aneurysmal perfusion and enlargement, brachial artery haematoma, splenic artery dissection, liver abscess, infected pseudocyst or pancreatitis. Seven patients required one or more additional interventions. Persistent aneurysmal perfusion, as in our case, represented the most common complication of elective treatment, occurring in 11% of patients in that series [5]. Thirty-three percent of patients (8 patients) in the surgical group had complications, of whom four required re-interventions. Complications included graft thrombosis and ileus, pseudoaneurysm, bile leak, intra-abdominal abscess and wound infections. Re-interventions were sometimes complex, including liver re-transplantation due to bile leak [5].

Currently, there is no clear consensus on the size of SMAAs that should be treated in asymptomatic patients. Surgical treatment of SMAA is clearly indicated in cases of rupture and infectious pathogenesis [8], but indications for endovascular or open surgical repair of asymptomatic lesions remain unclear, since favourable long-term results of endovascular treatment were reported in both groups [3,5,9]. Published series include relatively low number of patients and it is often difficult to extract firm data for decision-making. The majority of publications showed similar survival and re-intervention rates in open and endovascular approaches, with shorter hospital stay and lower complication rate for those patients treated with the latter approach [2]. There are some data showing lower invasiveness of endovascular management, followed by shorter hospital stay (mean 4 *vs.* 17 days, p<0.01) [3], pointing towards endovascular management of non-ruptured SMAAs. A recent publication [10], including a thorough review of the literature, suggests an advantage of the minimal invasiveness of endovascular therapy that might not be warranted in complicated or major aneurysms. Promising results with covered stents in SMA are reported also in the PERICLES registry, reporting results of chimney and snorkel stent grafts in more than 500 patients [11].

According to some authors, the aneurysmal size is important to decide whether to treat patients, since aneurysms with diameters smaller than 20 mm are considered to have a low rupture rate [3], although one group reported aneurysm size as a non-reliable predictor of VAA rupture [12]. Aneurysmal diameter should probably be compared to the size of the SMA in order to determine the ratio of arterial dilatation. Moreover, pregnancy planning, as in our patient, can be one of the reasons for the treatment – there is a high rupture risk during pregnancy (with high mortality rates) [9] and renal and splenic artery aneurysms in women of childbearing age should be treated in some authors’ opinion [3].

In our case, the decision for an endovascular treatment was made by a multidisciplinary team that consisted of vascular surgeons, cardiologists and interventional radiologists. Possible advantages and disadvantages were taken in consideration, including patient’s preferences.

Endovascular management offers different options, covered stents being only one of them. Suitable location proximally and a relatively accessible angle of the SMA together with a wide-neck aneurysm without branches were the major reasons for choosing a covered stent.

Coiling is also an alternative, but it would also have been technically challenging, although a smaller access sheath would have been sufficient. The aneurysm was quite large, requiring many coils for a complete exclusion. The neck was wide, so only stenting prior to coiling would have ensured safe coiling without distal embolization or SMA obstruction. Altogether, the procedure would have been quite complex, expensive, with long procedure time and considerable amount of irradiation we wanted to avoid.

Our case showed that distorted anatomy due to initial single femoral approach and a stiff guidewire resulted in inadequate positioning of the covered stent and therefore there was a need for re-intervention. Placing a large sheath into the SMA orifice, allowing for angiographic control just prior to deployment of the covered stent, would have probably resulted in an adequate positioning, although it might have increased the risk of complications (SMA dissection, access site haematoma etc.). An “access from above” (brachial access) in patients treated for symptomatic isolated dissection of the SMA proved to be more successful in comparison to the femoral approach, and in some patients it was used as a rescue procedure after a failed SMA cannulation [13]. Some authors conclude that selection of an arterial approach should be based on the morphology of the SMA arch. This is initially difficult to determine due to additional distortion made by the introduction of large sheaths, stiff guidewires and rigid or covered stents.

Alternatively, an additional access site could be chosen, providing space for angiographic catheter necessary for angiographic control. The “access from above” was not selected initially due to a relatively small access vessel and a large sheath needed. Our case showed that “access from above” was more suitable and should probably be used in the majority of cases with acceptable sizes of access vessels.

Median nerve palsy in our patient was probably a complication of the transaxillary access but was mild and temporary and did not influence long-term morbidity.

The aetiology of aneurysm in our patient remained unclear. A large majority of all detected aneurysms (60%) are mycotic by aetiology [6]. Due to young age and unclear aetiology of the aneurysm in our patient (except for family history), infection could be the cause. No signs of infection, as depicted in a publication [3], (fever, leucocytosis, pain, positive blood or tissue cultures or characteristic radiological findings) were detected in our patient.

The current follow-up protocol proposed for endovascular management of visceral aneurysms includes imaging at 3-month intervals during the first year and annually thereafter [5]. In our case, the SMA is easily accessible by US that will be performed each year. CTA will be used only in unclear US findings and if clinical symptoms occur.

## Conclusions

Endovascular management of an SMA aneurysm was safe and efficient in our case even after initial therapeutic failure. “Access from above” is probably safer and should be considered in the majority of cases with acceptable sizes of access vessels. Mid-term results of endovascular management in our patient are good, although life-long follow-up is planned to prevent late complications.
